# JNK pathway is involved in the inhibition of inflammatory target gene expression and NF-kappaB activation by melittin

**DOI:** 10.1186/1476-9255-5-7

**Published:** 2008-05-29

**Authors:** Hye Ji Park, Hwa Jeong Lee, Myung Sook Choi, Dong Ju Son, Ho Sueb Song, Min Jong Song, Jeong Min Lee, Sang Bae Han, Youngsoo Kim, Jin Tae Hong

**Affiliations:** 1 College of Pharmacy, Chungbuk National University, 12 Gaesin-dong, Heungduk-gu, Cheongju, Chungbuk 361-763, Korea; 2 College of Oriental Medicine, Kyungwon University, 65 Bukjeong-Dong, Sujeong-gu, Seongnam, Gyeonggi 461-701, Korea; 3 Department of Obstetrics and Gynecology, St. Vincent's Hospital, The Catholic University, Suwon 442-723, Korea; 4 Life Science R&D Center, Sinil Pharmaceutical Co, San 5-1, Bonpyung-Ri, Angsung-Myun, Chungju, Chungbuk,380-862, Korea

## Abstract

**Background:**

Bee venom therapy has been used to treat inflammatory diseases including rheumatoid arthritis in humans and in experimental animals. We previously found that bee venom and melittin (a major component of bee venom) have anti-inflammatory effect by reacting with the sulfhydryl group of p50 of nuclear factor-kappa B (NF-κB) and IκB kinases (IKKs). Since mitogen activated protein (MAP) kinase family is implicated in the NF-κB activation and inflammatory reaction, we further investigated whether activation of MAP kinase may be also involved in the anti-inflammatory effect of melittin and bee venom.

**Methods:**

The anti-inflammatory effects of melittin and bee venom were investigated in cultured Raw 264.7 cells, THP-1 human monocytic cells and Synoviocytes. The activation of NF-κB was investigated by electrophoretic mobility shift assay. Nitric oxide (NO) and prostaglandin E_2 _(PGE_2_) were determined either by Enzyme Linked Immuno Sorbent Assay or by biochemical assay. Expression of IκB, p50, p65, inducible nitric oxide synthetase (iNOS), cyclooxygenase-2 (COX-2) as well as phosphorylation of MAP kinase family was determined by Western blot.

**Results:**

Melittin (0.5–5 μg/ml) and bee venom (5 and 10 μg/ml) inhibited lipopolysaccharide (LPS, 1 μg/ml) and sodium nitroprusside (SNP, 200 μM)-induced activation of c-Jun NH2-terminal kinase (JNK) in RAW 264.7 cells in a dose dependent manner. However, JNK inhibitor, anthra [1,9-cd]pyrazole-6 (2H)-one (SP600215, 10–50 μM) dose dependently suppressed the inhibitory effects of melittin and bee venom on NF-κB dependent luciferase and DNA binding activity via suppression of the inhibitory effect of melittin and bee venom on the LPS and SNP-induced translocation of p65 and p50 into nucleus as well as cytosolic release of IκB. Moreover, JNK inhibitor suppressed the inhibitory effects of melittin and bee venom on iNOS and COX-2 expression, and on NO and PGE_2 _generation.

**Conclusion:**

These data show that melittin and bee venom prevent LPS and SNP-induced NO and PGE_2 _production via JNK pathway dependent inactivation of NF-κB, and suggest that inactivation of JNK pathways may also contribute to the anti-inflammatory and anti-arthritis effects of melittin and bee venom.

## Background

Bee venom therapy has been used to relieve pain and to treat inflammatory diseases including rheumatoid arthritis in humans [1] and in experimental animals [2]. Bee venom contains melittin, a 26 amino acid peptide, which forms an amphipathic helix with a highly charged carboxyl terminus [3]. We previously found that bee venom and its major component, melittin inhibited lipopolysaccharide (LPS), tumor necrosis factor-α (TNF-α), and sodium nitroprusside (SNP)-induced NF-κB activation by preventing p50 translocation through interaction of melittin and sulfhydryl residue of p50 and/or IκB kinases (IKKα and IKKβ), and that these inhibit inflammatory reaction in the development of rheumatoid arthritis [4,5] through reduction of large amounts of nitric oxide (NO) and prostaglandins (PGs) which are synthesized systemically in animal models of arthritis and in patients with rheumatoid arthritis [6-10].

NF-κB and IKKs have been suggested to play important roles in the regulation of inflammatory genes, such as, inducible nitric oxide synthetase (iNOS), cyclooxygenase-2 (COX-2), cytosolic phospholipase A_2 _(cPLA_2_), and tumor necrosis factor-α (TNF-α). Functionally active NF-κB exists mainly as a heterodimer consisting of subunits of the Rel family, and this heterodimer is normally sequestered in the cytosol as an inactive complex by binding to inhibitory κB (IκBs) in unstimulated cells [11]. The mechanism of NF-κB activation involves the phosphorylation of IκBs via IKK activation [12]. Once IκBs are phosphorylated, they are targeted for ubiquitination and subsequent degradation by the 26s proteosome [13]. The resulting free NF-κB is translocated to the nucleus, where it binds to the κB binding sites in the promoter regions of target genes, thereby controls their expression [14]. In several studies, potent inhibitors of IKKs preventing NF-κB activity through blockage of IκB release can be useful for the treatment of inflammatory diseases such as rheumatoid arthritis (RA) [15-18].

Mitogen activated protein (MAP) kinases are a group of signaling molecules that also appear to play important roles in inflammatory processes. At least three MAP kinase cascades; ERK (extracellular signal-regulated kinase), JNK (c-Jun N-terminal kinase) and p38 are well described, and have been reported to differentially activate depending on the stimuli and cell types [19]. Several studies have demonstrated that activation of MAP kinase is significant in the regulation of inflammation via controlling the activation of NF-κB and IKKs [19-22].

In the present study, we therefore investigated whether melittin and bee venom inhibit NF-κB via disrupting MAP kinase signals, and thereby inhibit the inflammatory response in Raw 264.7 macrophages and in the synoviocytes of rheumatoid arthritis patients.

## Methods

### Chemicals

Rabbit polyclonal antibodies to cPLA_2 _(dilution 1:500), and goat polyclonal antibody to COX-2 (1:500), TNF-α (1:500), p50 (1:500), p65 (1:500), IκBα (1:500), phospho-IκBα (1:200), IκBβ (1:500) and mouse polyclonal antibody to iNOS (1:500), IκB kinases (1:500), mouse monoclonal phospho-ERK, phospho-JNK and phospho-p38 antibodies (1:500), and rabbit polyclonal ERK, JNK and p38 (1;500), and all of the secondary antibodies used in Western blot analysis were purchased from Santa Cruz Biotechnology (Santa Cruz, CA, USA). T4 polynucleotide kinase was obtained from Promega (Madison, WI). Poly (dI·dC), horseradish peroxidase-labeled donkey anti-rabbit second antibody, and the ECL detection reagent were obtained from Amersham Pharmacia Biotech (Centennial Ave, NJ, USA). SNP, LPS, Griess reagent, monoclonal anti-β-actin antibody, 3-(4,5-dimethyl-2-thiazolyl)-2,5-diphenyl tetrazolium bromide (MTT) and melittin, a component of bee venom were purchased from Sigma-Aldrich (St. Louis, MO, USA). U0126 (ERK inhibitor, 1,4-diamino-2,3-dicyano-1,4-bis (2-aminophenylthio)butadiene) and SP600125 (JNK inhibitor, anthra [1,9-cd]pyrazole-6 (2H)-one) were purchased from Calbiochem (San Diego, CA, USA). Bee venom was purchased from You-Miel BV Ltd (Hwasoon, Korea). The compositions are followings: melittin (45–50%), apamin (2.5–3%), MCD peptide (2–3%), PLA2 (12%), Lyso PLA (1%), hyaluronidase (2–3%), histidine (1–1.5%), secarpin (0.5%), tertiapin (0.1%), procamine (0.1%), amine (2–3%), carbohydrate (4–5%), 6pp lipids (4–5%), and others (19–27%, protease inhibitor, glucosidase, invertase, acid phosphomonoesterase, dopamine, norepinephrine and unknown amino acid).

### Cell culture

Raw 264.7, a mouse macrophage-like cell line and THP-1, a human monocytic cell line were obtained from the American Type Culture Collection (Cryosite, Lane Cove NSW, Australia). Dulbecco's modified Eagle medium (DMEM), penicillin, streptomycin, and fetal bovine serum were purchased from Gibco Life Technologies (Rockville, MD, USA). Raw 264.7 cells were grown in DMEM with 10% fetal bovine serum, 100 U/ml penicillin, and 100 μg/ml streptomycin at 37°C in 5% CO_2 _humidified air. THP-1 cells were grown in RPMI 1640 with L-glutamine and 25 mM HEPES buffer (Gibco Life Technologies, Rockville, MD, USA) supplemented with 10% fetal bovine serum, 100 units/ml penicillin and 100 μg/ml streptomycin at 37°C in 5% CO_2 _humidified air.

### Synoviocyte culture

Synovial tissues were obtained, with consent, from nine RA patients who were undergoing total knee replacement or arthroscopic synovectomy. All patients satisfied the 1987 revised diagnostic criteria of the American College of Rheumatology [23]. The method of synoviocyte culture was described in elsewhere [4,5].

### Determination of Nitric Oxide and Prostaglandin E_2_

The NO accumulation in the supernatant was assessed by Griess reaction described in elsewhere [4], and the determination of PGE_2 _was performed as described in elsewhere [4].

### DNA binding activity of NF-κB

EMSA was performed according to the manufacturer's recommendations (Promega, Madison, WI) as described in previous study [4,5]. Briefly, nuclear extract was incubated with κB consensus oligonucleotides end-labeled using T4 polynucleotide kinase and [γ-32P] ATP for 10 min at 37°C. Gel shift reactions were assembled and allowed to incubate at room temperature for 10 min followed by the addition of 1 μl (50,000–200,000 cpm) of 32P-labeled oligonucleotide and another 20 min of incubation at room temperature. For the competition assay, 100× or 200× excesses of unlabeled double-stranded oligonucleotide of the κB binding site (or 100× irrelevant oligonucleotide of AP-1 or SP-1) were used as specific competitors. Supershift assay was done in the presence of p50 or p65 subunit of NF-κB (2 μg). Subsequently 1 μl of gel loading buffer was added to each reaction and loaded onto a 4% nondenaturing gel and electrophoresed until the dye was three-fourths of the way down the gel. The gel was dried at 80°C for 1 hr and exposed to film overnight at 70°C. The relative density of the protein bands was scanned by densitometry using MyImage (SLB, Seoul, Korea), and quantified by Labworks 4.0 software (UVP Inc., Upland, California).

### Transfection and assay of Luciferase activity

Raw 264.7 or THP-1 cells were transfected with pNF-κB-Luc plasmid (5× NF-κB; Stratagene, CA, USA) using a mixture of plasmid and lipofectAMINE PLUS in OPTI-MEN according to manufacture's specification (Invitrogen, Carlsbad, CA, USA). The control pCMV (Clontech, CA, USA) was co-transfected to monitor the transfection efficiency. After 24 hr, the cells were then co-treated with BV (or melittin) and LPS or SNP. Luciferase activity was measured by using the luciferase assay kit (Promega) according to the manufacturer's instructions (WinGlow, Bad Wildbad, Germany).

### Western blot analysis

Cell lysates were prepared as described in the previous study [12]. Equal amount of lysate proteins (80 μg) were separated on a SDS/12%-polyacrylamide gel, and then transferred to a nitrocellulose membrane (Hybond ECL, Amersham Pharmacia Biotech Inc., Piscataway, NJ). Blots were blocked for 2 hr at room temperature with 5% (w/v) non-fat dried milk in Tris-buffered saline [10 mM Tris (pH 8.0) and 150 mM NaCl] solution containing 0.05% tween-20. The membrane was incubated for 5 hr at room temperature with specific antibodies. The blot was then incubated with the corresponding conjugated anti-rabbit immunoglobulin G-horseradish peroxidase (Santa Cruz Biotechnology Inc.). Immunoreactive proteins were detected with the ECL western blotting detection system. The relative density of the protein bands was scanned by densitometry using MyImage (SLB, Seoul, Korea), and quantified by Labworks 4.0 software (UVP Inc., Upland, California).

### Immunofluorescence staining

Cells were plated in chambered tissue culture slides at a density of 2 × 10^3 ^cells/well in DMEM. The Raw 264.7 cells were then cultured with serum-free medium containing LPS, BV and SP for 2 hr, and then the intracellular location of p50 was determined by immunofluorescence confocal scanning microscope (magnification, 630×) as described in elsewhere [4]. Twenty-four hours later, the cells were washed once with PBS and fixed with 4% paraformaldehyde for 20 min, membrane-permeabilized by exposure for 2 min to 0.1% Triton ×-100 in phosphate-buffered saline, and placed in blocking serum (5% bovine serum albumin in phosphate-buffered saline) at room temperature for 1 hr. The cells were then exposed to primary polyclonal antibodies for p50 (1:100 dilution) overnight at 4°C, After washes with ice-cold PBS followed by treatment with anti-goat- or anti- rabbit- biotinylated secondary antibodies Alexa Fluor 568 (p50) or Alexa Fluor 633 (DAPI) (Molecular Probes Inc., Eugene, OR, USA), 1:200 dilution, for 4 hr at room temperature. Nuclear stain and mount in antifade medium with DAPI (Vector Laboratory Inc.), immunofluorescence images were acquired using a confocal laser scanning microscope (TCS SP2, Leica Microsystems AG, Wetzlar, Germany) equipped with a 630×oil immersion objective.

### Statistical analysis

Data were analyzed using one-way analysis of variance followed by Tukey's test as a post hoc test. Differences were considered significant at p < 0.05.

## Results

### Melittin inhibited LPS and SNP-induced activation of JNK in RAW 264.7 cells

**Figure 1:**
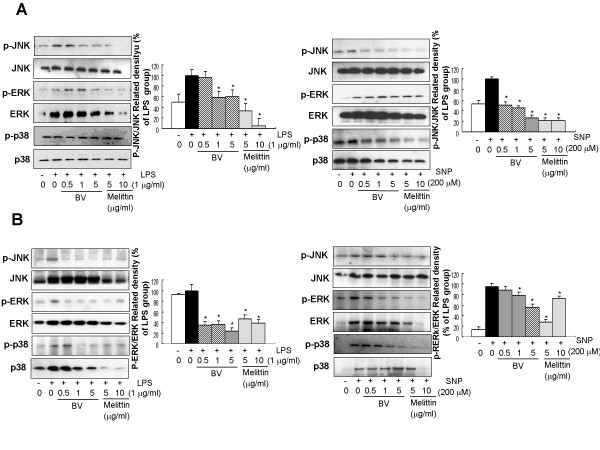
**Effect of melittin and bee venom on LPS and SNP-induced phosphorylation of MAPKs**. **A**, Raw 264.7 macrophages were treated with 5 or 10 μg/ml melittin or 0.5–5 μg/ml bee venom in the presence of LPS (1 μg/ml) or SNP (200 μM) at 37°C for 24 hr. **B**, Synoviocytes were treated with 5 or 10 μg/ml melittin or 0.5–5 μg/ml bee venom in the presence of 1 μg/ml LPS or 200 μM SNP at 37°C for 24 hr. Equal amounts of total proteins (80 μg/lane) were subjected to 10% SDS ± PAGE, and the expression of p-ERK/ERK, p-JNK/JNK, or p-p38/p38 were detected by western blotting using specific antibodies. Each panel representative of three independent experiments. Quantification of band intensities from three independent experimental results was determined by a densitometry (Imaging System). Data was described as means ± S.E. from three experiments performed in triplicate for p-ERK/ERK, p-JNK/JNK, or p-p38/p38. *p < 0.05 indicate statistically significant differences from the LPS or SNP-treated group.

We previously found that bee venom and its major component, melittin inhibits LPS, TNF-α and SNP-induced inflammatory responses through inactivation of NF-κB and IKKs signals [4,5]. The MAPK pathway is known to play an important role in the transcriptional regulation of LPS-induced iNOS and COX-2 expression via suppression of the activation of transcription factor NF-κB. To investigate the involvement of MAP kinase pathway in the inhibitory effect by melttin and bee venom on NO and PGE_2 _production, the activation of MAP kinase (phosphorylation of ERK, JNK and p38) induced by LPS and SNP was evaluated in both Raw 264.7 cells as well as synoviocytes. The densitometry analysis from individual three different experiments showed that melittin (0.5–5 μg/ml) and bee venom (5 and 10 μg/ml) strongly blocked LPS (1 μg/ml) and SNP (200 μM)-induced activation of JNK in the Raw 264.7 cells (Fig. 1A) as well as synoviocytes (Fig. 1B). We also found that significant inhibitory effects of melittin (0.5–5 μg/ml) on the activation of ERK in LPS treated Raw 264.7 cells and synoviocytes, and SNP treated synoviovytes. Activation of p38 was also significantly reduced in the LPS treated synoviocytes, and SNP treated Raw 264.7 cells and synoviocytes, but the expression ERK and p38 was also reduced, indicating that blocking of the activation of p38 and ERK was not specific (Fig. 1A and 1B). Similar effect of bee venom was also found (Fig. 1). These results suggest that JNK could be the most specific and important signal involved in the melittin and BV-induced inhibition of NO and PGE_2 _generation.

### JNK inhibitor suppressed the inhibitory effects of melittin and bee venom on NF-κB dependent Luciferase and DNA binding activity

**Figure 2:**
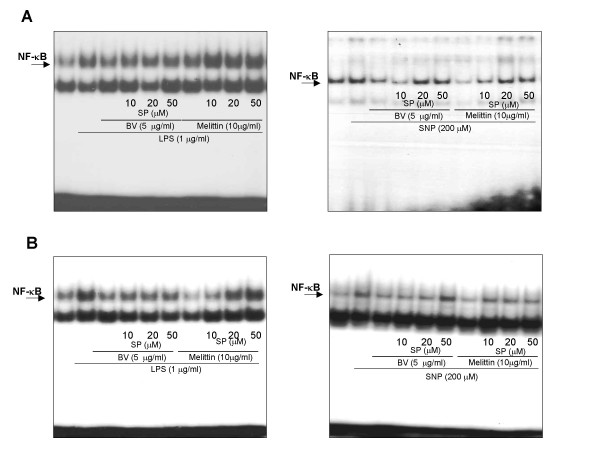
**JNK inhibitor suppressed the inhibitory effect of melittin and bee venom on the NF-κB DNA binding activity induced by LPS or SNP**. Raw 264.7 macrophages (**A**) and synoviocytes (**B**) were pretreated with 10, 20, and 50 μM SP600125 1 h prior to the treatment with melittin or bee venom with or without LPS or SNP for 2 h. The DNA binding activation of NF-κB was investigated using EMSA. Nuclear extracts from Raw 264.7 cells or synoviocytes treated for 1 hr were incubated with ^32^P-end-labeled oligonucleotide containing the κB sequence. Each panel is representative of three similar experiments with duplicates.

To further examine the involvement of JNK pathway in the inhibitory effect of melittin and bee venom on NF-κB activation, we explored JNK specific inhibitor SP600125 (10–50 μM), and determined the inhibitory effect of melittin and bee venom on the activation of NF-κB. As shown in Fig. 2, pretreatment (1 hr) of SP600125 strongly suppressed the inhibitory effect of melittin and bee venom on the LPS and SNP-induced NF-κB activation in Raw 264.7 cells (Fig. 2A) and synoviocytes (Fig. 2B). The specificity of DNA binding was examined by competition assay by adding an excessive amount of unlabeled/cold oligonucleotides to reaction mixtures containing nuclear extract. The specificity of DNA binding was examined by supershift assay using antibodies for the p50 or p65 components of NF-κB (data not shown).

**Figure 3:**
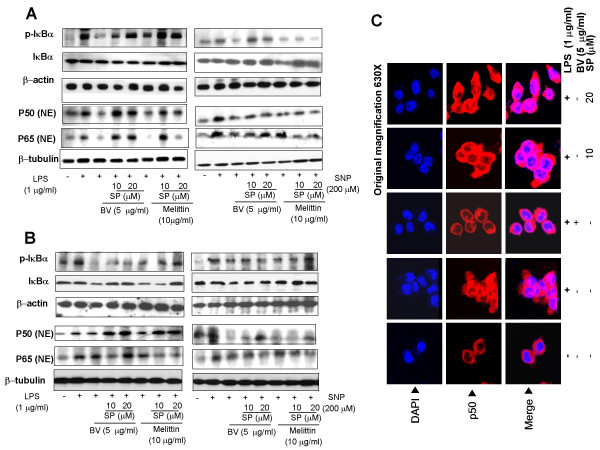
JNK inhibitor suppressed the inhibitory effect of melittin and bee venom on the nuclear translocation of the p50 subunit and the release of IκB induced by LPS or SNP. Raw 264.7 cells (A) or synoviocytes (B) were pretreated with 10 and 20 μM SP600125 1 h prior to the treatment with melittin or bee venom with or without LPS (1 μg/ml) or SNP (200 μM) at 37°C for 24 hr. 80 μg of nuclear (p50 and p65), cytosolic IκB or total protein extracted after treatment were used to determine of p50, p65, p-IκBα, IκBα, or IκBβ; β-actin protein was used as an internal control. Each panel is representative of three similar experiments. C, Raw 264.7 cells were treated with LPS, BV and SP for 24 hr, and then the intracellular location of p50 was determined by immunofluorescence confocal scanning microscope (magnification, 630×). Double staining (Merge, pink) with p50 (red) and DAPI (blue) staining demonstrates the localization of p50 in the nucleus.

One of the consequences of inhibition of NF-κB is the inhibition of the nuclear translocation of p50 and p65 through the blockage of IκB release. To study the result of the treatment of JNK inhibitor on the translocation of p50 and p65 into the nucleus, we determined the appearance of the p50 and p65 in the nucleus extracts by Western blott. Pretreatment (1 hr) of SP600125 suppressed the inhibitory effect of melittin and bee venom on LPS and SNP-induced nuclear translocation of the p50 and p65 in Raw 264.7 cells (Fig. 3A) and in synoviocytes (Fig. 3B). Te kinetics of IκBα release (determined the level of IκBα phosphorylation) in cytosol were further studied by western blot analysis. Inhibitory effect of melittin and bee venom on the LPS as well as SNP-induced IκBα release was markedly suppressed by SP600125 in both Raw 264.7 cells (Fig. 3A) and synoviocytes (Fig. 3B). The phosphorylation of IκBβ was not examined because this antibody is not commercially available. The suppressive effect of SP600125 on the reduced nuclear translocation of the p50 subunits was also confirmed by examination with confocal laser scanning microscopy in Raw 264.7 cells (Fig. 3C).

**Figure 4:**
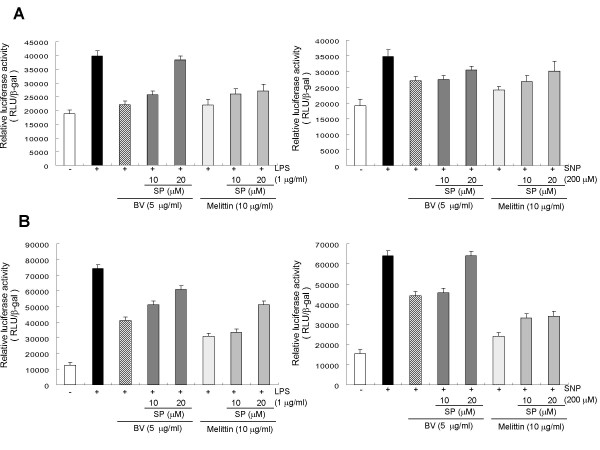
JNK inhibitor suppressed the inhibitory effect of melittin and bee venom on the on NF-κB-dependent luciferase induced by LPS or SNP. Raw 264.7 cells (A) and THP-1 cells (B) were transfected with pNF-κB-Luc plasmid (5× NF-κB), Raw 264.7 cells or THP-1 cells were pretreated with 10 and 20 μM SP600125 1 hr prior to the treatment with melittin or bee venom with or without LPS (1 μg/ml) or SNP (200 μM) at 37°C for 2 hr, and then luciferase activities were determined. All values represent means ± S.E. of three independent experiments performed in triplicate.

Raw 264.7 and THP-1 cells were transfected with a promoter reporter gene construct (a fusion gene containing SV40 promoter, 5 repeats of the consensus NF-κB binding sequence), and transcriptional activities were also measured after stimulating the cells with LPS or SNP in the presence of bee venom and melittin. Agreement with the suppressive effect of SP600125 on the DNA binding activity of NF-κB, pretreatment (1 hr) of SP600125 also strongly suppressed the inhibitory effect of melittin and bee venom on the LPS or SNP-induced NF-κB transcriptional activation in Raw 264.7 cells (Fig. 4A) and THP-1 cells (Fig. 4B). These suppressive effects were statistically significant in the inhibitory effect of melittin and Bee venom on the LPS and SNP-induced NF-κB transcriptional activation in both Raw 264.7 cells (Fig. 4A) and THP-1 cells (Fig. 4B) by 20 μM of SP600125. The suppressive effects were also statistically significant in the inhibitory effect of Bee venom on the LPS-induced, and in the inhibitory effect of melittin on the SNP-induced NF-κB in THP-1 cells by 10 μM of SP600125.

### JNK inhibitor suppressed the inhibitory effects of melittin and bee venom on iNOS and COX-2 expression, and on NO and PGE_2 _generation

**Figure 5:**
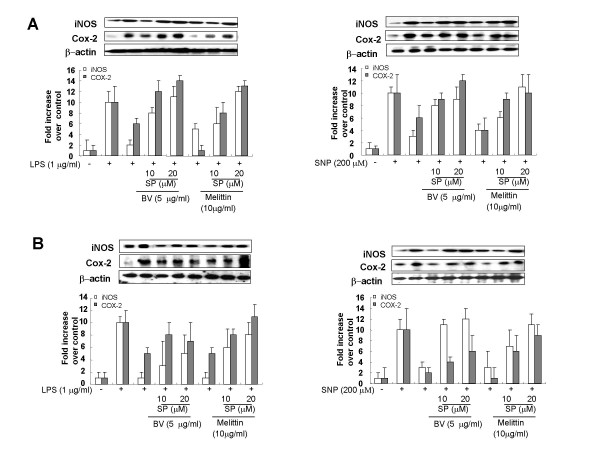
**JNK inhibitor suppressed inhibitory effect of melittin and bee venom on the inflammatory gene expression induced by LPS or SNP**. Raw 264.7 cells (**A**) or synoviocytes (**B**) were pretreated with 10 and 20 μM SP600125 1 hr prior to the treatment with melittin or bee venom with or without LPS (1 μg/ml) or SNP (200 μM) at 37°C for 24 hr. Equal amounts of total proteins (80 μg/lane) were subjected to 10% SDS ± PAGE, and the expression of iNOS, COX-2 and β-actin were detected by western blotting using specific antibodies. Each panel representative of three independent experiments.

**Figure 6:**
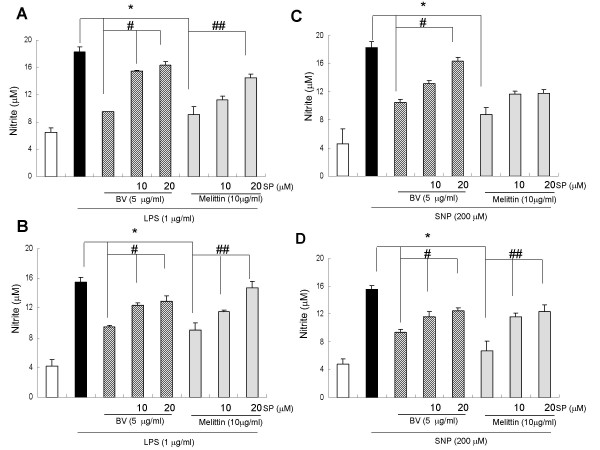
**JNK inhibitor suppressed the inhibitory effect of melittin and bee venom on the generation of NO induced by LPS or SNP**. Raw 264.7 cells (**A, C**) or synoviocytes (**B, D**) were pretreated with 10 and 20 μM SP600125 1 hr prior to the treatment with melittin or bee venom with or without LPS (1 μg/ml) or SNP (200 μM) at 37°C for 24 hr. The amounts of NO in the medium of cultured Raw264.7 cells (A, C) or synoviocytes (B, D) were determined by the methods described in the methods. Results are expressed as means ± SE of three independent experiments performed in triplicate. * indicates significantly different from the LPS or SNP treated groups (p < 0.05). ^# ^and ^##^, indicates significantly different from LPS or SNP + melittin or BV treated group (p < 0.05).

**Figure 7:**
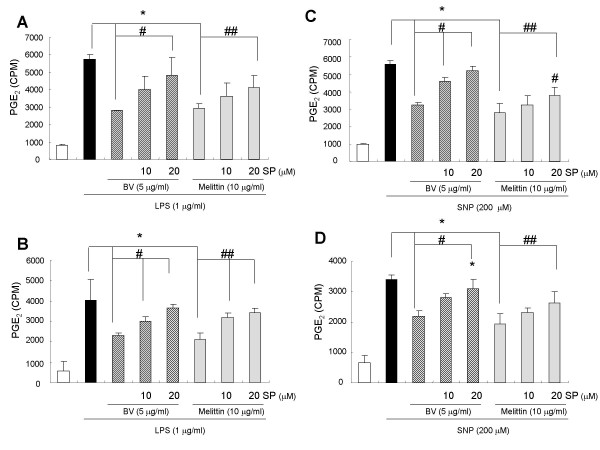
**JNK inhibitor suppressed the inhibitory effect of melittin and bee venom on the generation of PGE_2 _induced by LPS or SNP**. Raw 264.7 cells (**A, C**) or synoviocytes (**B, D**) were pretreated with 10 and 20 μM SP600125 1 hr prior to the treatment with melittin or bee venom with or without LPS (1 μg/ml) or SNP (200 μM) at 37°C for 24 hr. The amounts of PGE_2 _in the medium of cultured Raw264.7 cells (A, C) or synoviocytes (B, D) were determined by the methods described in the methods. Results are expressed as means ± SE of three independent experiments performed in triplicate. * indicates significantly different from the LPS or SNP treated groups (p < 0.05). ^# ^and ^##^, indicates significantly different from LPS or SNP + melittin or BV treated group (p < 0.05).

To investigate whether the suppressed effect of SP600125 on the inhibitory effect of bee venom and melittin on the inflammatory gene expression, iNOS and COX-2 expression was determined. The inhibitory effect of melittin and bee venom on iNOS and COX-2 expression by LPS and SNP in Raw 264.7 cells (Fig. 5A) and in synoviocytes (Fig. 5B) were dose dependently suppressed by SP600215 (10 and 20 μM). The suppressive effect of SP600125 on the inflammatory mediator generation was then examined. Significant concentration-dependent suppression by the pretreatment of SP600215 on the NO generation was observed in Raw 264.7 cells (Fig. 6A,C) and synoviocytes (Fig. 6B,D) treated with melittin and bee venom in combination with LPS (Fig. 6A,B) and SNP (Fig. 6C,D). Significant concentration-dependent suppressive effect by the pretreatment of SP600215 (10 and 20 μM) on the PGE_2 _generation was also observed in Raw 264.7 cells (Fig. 7A,C) and synoviocytes (Fig. 7B,D) treated with melittin and bee venom in combination with LPS (Fig. 7A,C) and SNP (Fig. 7B,D).

## Discussion

We previously found that bee venom and its major component, melittin inhibits inflammatory stimuli such as LPS, TNF-α, and SNP-induced NF-κB activation by preventing p50 translocation via an interaction between melittin and sulfhydryl group of p50 and/or IKKα and IKKβ, and that these inhibit inflammatory reaction in the development of rheumatoid arthritis [4,5]. In the present study, we further found that melittin and bee venom significantly reduced inflammatory stimuli (LPS and SNP)-induced activation of JNK signal, and the JNK signal specific inhibitor SP600215 suppressed the inhibitory effect of melittin and bee venom on the NF-κB activation, and inflammatory reaction in Raw 264.7 macrophages and synoviocytes obtained from rheumatoid arthritis patients. This data reflected that the inhibition of JNK pathway conjunction with inhibition of NF-κB pathway may also contribute to the inhibitory effect of melittin and bee venom on the inflammatory reaction of arthritis rheumatism.

LPS and SNP rapidly phosphorylates ERK, p38 and JNK, which lead to NF-κB activation in macrophages [24,25]. The activation of this MAP kinase leads an increase in the production of pro-inflammatory mediators such as NO and PGE_2 _[26,27]. Several studies have demonstrated the implication of the activation of MAP kinase in LPS-induced iNOS and COX-2 expression [28-30] and the activation of NF-κB [30-33]. To demonstrate other pathway of NF-κB inactivation by melittin and bee venom, we investigated the relationship between NF-κB and MAP kinase activation. Our data demonstrated that melittin and bee venom reduces LPS and SNP-induced activation of JNK signals. Even though other signals (p38 MAP kinase and ERK signal) may be also interfered by melittin and bee venom depend on the cell types and stimuli, LPS and SNP-induced JNK signal was specifically inhibited by melittin and bee venom. This finding is agreed with other data showing that JNK pathway is important signal in the activation of NF-κB in the processes of inflammatory reaction [29,30,34]. In more precise investigation with specific JNK inhibitor SP600215, we further showed that the combination treatment of JNK inhibitor with bee venom and melittin suppressed inhibitory effects of melittin and bee venom on the LPS and SNP-induced NO and PGE_2 _release with the suppressed effect on the inhibitory effect of melittin and bee venom on the NF-κB DNA binding and transcriptional activities. Moreover, we also showed that JNK inhibitor SP600215 abrogated the inhibitory effect of melittin and bee venom on the LPS and SNP-induced translocation of NF-κB by western blotting as well as translocation of p50, a subunit of NF-κB by confocal microscope observation. These data show that specific inhibition of JNK pathway may be important for inactivation of NF-κB, and thus inhibitory effects of melittin and bee venom on the LPS and SNP-induced NO and PGE_2 _production.

The involvement of MAPK pathways in the biological activities of melittin and bee venom has been demonstrated. Bee venom triggered the activation of p38 MAPK and JNK and increased lactate dehydrogenase (LDH) release in the bee venom-induced apoptosis of human leukemic U937 [35]. Very similar to our finding, Jang et al. showed that bee venom inhibited mRNA level of iNOS, COX-2 and NF-κB paralleled with inhibition of mRNA level of MAP kiase induced by LPS [36]. In addition, we also found that bee venom and melittin inhibited platelet-derived growth factor BB (PDGF-BB)-induced smooth muscle cell proliferation through inactivation of NF-κB via inhibition of ERK pathway [37]. These results suggest that, the cross talking between the MAP kinase and the NF-κB signals may be important for relaying the biological effect of melittin and bee venom. Several studies have been reported the cross talking between MAP kinase signals and NF-κB signals. Minutoli et al., demonstrated the abrogation of JNK and p38 signals, but enhancement of ERK 1/2 activity by disruption of the transcriptional factor NF-κB in the development of testicular ischemia-reperfusion injury [38]. It was also found that TNF-induced NF-κB activation was abrogated in cells deleted of MKK4 gene which is a dual-specificity kinase that activates both JNK and p38 MAPK [39].

Differential MAPK pathways in the activation of NF-κB can be activated depend upon cell types and stimuli as well as biological activities. It is noteworthy that a NF-κB inducing kinase activates NF-κB transcriptional activity through a p38 MAPK-dependent RelA phosphorylation pathway in the induction of pro-inflammatory gene expression [40]. However, agreement with our finding, de Haij et al., demonstrated that NF-κB mediated IL-6 production by renal epithelial cells in the tubulointerstitial inflammation, a hallmark of most renal diseases is regulated by JNK [41]. JNK pathway is also involved in IL-6 gene expression by enhancing NF-κB activity in human monocytes [42], as well as induction of proinflammatory responses in macrophages by the glycosylphosphatidylinositols of Plasmodium falciparum [43]. Taken together, our data indicate that inhibition of JNK signal is the most involved in the inhibitory effect of melittin and bee venom on the LPS and SNP-induced activation of NF-κB as well as in the production of NO and PGE_2_. In inflammatory diseases, PGs and NO contribute to the pathophysiology of local and chronic inflammation [44-47]. The promoter region of the murine gene encoding iNOS and COX-2 contains NF-κB binding sites [15,48], which suggests that the inhibitory effect of inflammatory gene expression is related with the inhibition of the DNA binding activity of NF-κB. The therapeutic potential of the inhibition of NF-κB activity has been recognized as an effective anti-inflammatory treatment strategy against the progression of arthritis [49]. Therefore, the present data show that inhibition of JNK pathway may also contribute to the anti-inflammatory and anti-arthritis effects of melittin and bee venom, and cross talking between JNK and NF-κB signals may be important anti-inflammatory mechanism of melittin and bee venom.

## Conclusion

These data show that melittin and bee venom prevent LPS and SNP-induced NO and PGE_2 _production via JNK pathway dependent inactivation of NF-κB, and suggest that inactivation of JNK pathways may also contribute to the anti-inflammatory and anti-arthritis effects of melittin and bee venom.

## Abbreviations

COX-2: cyclooxgenase-2; cPLA_2_: cytosolic phospholipase A2; DTT: dithiothreitol; ELISA: Enzyme Linked Immuno Sorbent Assay; EMSA: electrophoretic mobility shift assay; ERK: extracellular signal-regulated kinase; GSH: glutathione; iNOS: inducible nitric oxide synthetase; IKK: IκB kinase; IκB: inhibitory κB; JNK: c-Jun NH2-terminal kinase; LPS: lipopolysaccharide; NF-κB: nuclear factor-kappa B; NO: nitric oxide; PGE_2_: prostaglandin E_2_; SB203580: methylsulfinylphenyl)-5-(4-pyridyl)imidazole; SNP: sodium nitroprusside; SP600125: anthra [1,9-cd]pyrazole-6 (2H)-one; TNF-α: Tumor necrosis factor-α; U0126,1,4-diamino-2,3-dicyano-1,4-bis (2-aminophenylthio)butadiene.

## Competing interests

The authors declare that they have no competing interests.

## Authors' contributions

HJP carried out RAW264.7 cells culture, and participated in the NF-κB luciferase assay, HJL carried out western blot analysis, MSC carried out the DNA binding activity of NF-κB, JWL carried out Synoviocyte culture, DJS participated in Prostaglandin E_2 _assay, HSS KYS and LBS participated in study design and coordination as well as editing of the manuscript, JTH participated in the design of this study and prepared the manuscript. All authors have read and approved the final manuscript.

## References

[B1] Billingham ME, Morley J, Hanson JM, Shipolini RA, Vernon CA (1973). Letter: An anti-inflammatory peptide from bee venom. Nature.

[B2] Kwon YB, Lee JD, Lee HJ, Han HJ, Mar WC, Kang SK, Beitz AJ, Lee JH (2001). Bee venom injection into an acupuncture point reduces arthritis associated edema and nociceptive responses. Pain.

[B3] Perez-Paya E, Houghten RA, Blondelle SE (1995). The role of amphipathicity in the folding, self-association and biological activity of multiple subunit small proteins. J Biol Chem.

[B4] Park HJ, Lee SH, Son DJ, Oh KW, Kim KH, Song HS, Kim GJ, Oh GT, Yoon DY, Hong JT (2004). Antiarthritic effect of bee venom: inhibition of inflammation mediator generation by suppression of NF-kappaB through interaction with the p50 subunit. Arthritis Rheum.

[B5] Park HJ, Son DJ, Lee CW, Choi MS, Lee US, Song HS, Lee JM, Hong JT (2007). Melittin inhibits inflammatory target gene expression and mediator generation via interaction with IkappaB kinase. Biochem Pharmacol.

[B6] Salvemini D, Misko TP, Masferrer JL, Seibert K, Currie MG, Needleman P (1993). Nitric oxide activates cyclooxygenase enzymes. Proc Natl Acad Sci USA.

[B7] Guastadisegni C, Nicolini A, Balduzzi M, Ajmone-Cat MA, Minghetti L (2002). Modulation of PGE(2) and TNFalpha by nitric oxide and LPS-activated RAW 264.7 cells. Cytokine.

[B8] Pelletier JP, Jovanovic D, Fernandes JC, Manning P, Connor JR, Currie MG, Di Battista JA, Martel-Pelletier J (1998). Reduced progression of experimental osteoarthritis in vivo by selective inhibition of inducible nitric oxide synthase. Arthritis Rheum.

[B9] Yang Y, Hutchinson P, Morand EF (1999). Inhibitory effect of annexin I on synovial inflammation in rat adjuvant arthritis. Arthritis Rheum.

[B10] Longo WE, Panesar N, Mazuski J, Kaminski DL (1994). Contribution of cyclooxygenase-1 and cyclooxygenase-2 to prostanoid formation by human enterocytes stimulated by calcium ionophore and inflammatory agents. Prostaglandins Other Lipid Mediat.

[B11] Baeuerle PA (1998). IkappaB-NF-kappaB structures: at the interface of inflammation control. Cell.

[B12] O'Connell MA, Bennett BL, Mercurio F, Manning AM, Mackman NJ (1998). Role of IKK1 and IKK2 in lipopolysaccharide signaling in human monocytic cells. J Biol Chem.

[B13] Magnani M, Crinelli R, Bianchi M, Antonelli A (2000). The ubiquitin-dependent proteolytic system and other potential targets for the modulation of nuclear factor-κB (NF-κB). Curr Drug Targets.

[B14] Karin M, Delhase M (2000). The I kappa B kinase (IKK) and NF-kappa B: key elements of proinflammatory signalling. Semin Immunol.

[B15] Tak PP, Gerlag DM, Aupperle KR, Geest DA, Overbeek M, Bennett BL, Boyle DL, Manning AM, Firestein GS (2001). Inhibitor of nuclear factor kappaB kinase beta is a key regulator of synovial inflammation. Arthritis Rheum.

[B16] Jeon KI, Byun MS, Jue DM (2003). Gold compound auranofin inhibits IkappaB kinase (IKK) by modifying Cys-179 of IKKbeta subunit. Exp Mol Med.

[B17] Cuzzocrea S, Wayman NS, Mazzon E, Dugo L, Di Paola R, Serraino I, Britti D, Chatteriee pk, Caput AP, Thiemermann C (2002). The cyclopentenone prostaglandin 15-deoxy-Delta(12,14)-prostaglandin J(2) attenuates the development of acute and chronic inflammation. Mol Pharmacol.

[B18] Kapahi P, Takahashi T, Natoli G, Adams SR, Chen Y, Tsien RY, Karin M (2002). Inhibition of NF-kappa B activation by arsenite through reaction with a critical cysteine in the activation loop of Ikappa B kinase. J Biol Chem.

[B19] Moon DO, Park SY, Lee KJ, Heo MS, Kim KC, Kim MO, Lee JD, Choi YH, Kim GY (2007). Bee venom and melittin reduce proinflammatory mediators in lipopolysaccharide-stimulated BV2 microglia. Int Immunopharmacol.

[B20] Kim JH, Kim DH, Baek SH, Lee HJ, Kim MR, Kwon HJ, Lee CH (2006). Rengyolone inhibits inducible nitric oxide synthase expression and nitric oxide production by down-regulation of NF-kappaB and p38 MAP kinase activity in LPS-stimulated RAW 264.7 cells. Biochem Pharmacol.

[B21] Kim YH, Lee SH, Lee JY, Choi SW, Park JW, Kwon TK (2004). Triptolide inhibits murine-inducible nitric oxide synthase expression by down-regulating lipopolysaccharide-induced activity of nuclear factor-kappa B and c-Jun NH2-terminal kinase. Eur J Pharmacol.

[B22] Pergola C, Rossi A, Dugo P, Cuzzocrea S, Sautebin L (2006). Inhibition of nitric oxide biosynthesis by anthocyanin fraction of blackberry extract. Nitric Oxide.

[B23] Arnett FC, Edworthy SM, Bloch DA, McShane DJ, Fries JF, Cooper NS, Healey LA, Kaplan SR, Lianq MH, Luthra HS (1998). The American Rheumatism Association 1987 revised criteria for the classification of rheumatoid arthritis. Arthritis Rheum.

[B24] Cario E, Rosenberg IM, Brandwein SL, Beck PL, Reinecker HC, Podolsky DK (2000). Lipopolysaccharide activates distinct signaling pathways in intestinal epithelial cell lines expressing Toll-like receptors. J Immunol.

[B25] Zhang FX, Kirschning CJ, Mancinelli R, Xu XP, Jin Y, Faure E, Mantovani A, Rothe M, Muzio M, Arditi M (1999). Bacterial lipopolysaccharide activates nuclear factor-kappaB through interleukin-1 signaling mediators in cultured human dermal endothelial cells and mononuclear phagocytes. J Biol Chem.

[B26] Kuprash DV, Udalova IA, Turetskaya RL, Kwiatkowski D, Rice NR, Nedospasov SA (1999). Similarities and differences between human and murine TNF promoters in their response to lipopolysaccharide. J Immunol.

[B27] Rudders S, Gaspar J, Madore R, Voland C, Grall F, Patel A, Pellacani A, Perrella MA, Libermann TA, Oettgen P (2001). ESE-1 is a novel transcriptional mediator of inflammation that interacts with NF-κB to regulate the inducible nitric-oxide synthase gene. J Biol Chem.

[B28] Ban HS, Suzuki K, Lim SS, Jung SH, Lee SH, Ji J, Lee HS, Lee YS, Lee YS, Shin KH, Ohuchi K (2004). Inhibition of lipopolysaccharide-induced expression of inducible nitric oxide synthase and tumor necrosis factor-αby 2'-hydroxychalcone derivatives in RAW 264.7 cells. Biochem Pharmacol.

[B29] Chen BC, Chen YH, Lin WW (1999). Involvement of p38 mitogen-activated protein kinase in lipopolysaccharide-induced NOS 2 and COX-2 expression in J774 macrophages. Immunology.

[B30] Lee SY, Son DJ, Lee YK, Lee JW, Lee HJ, Yun YW, Ha TY, Hong JT (2006). Inhibitory effect of sesaminol glucosides on lipopolysaccharide-induced NF-kappaB activation and target gene expression in cultured rat astrocytes. Neurosci Res.

[B31] Tsao LT, Tsai PS, Lin RH, Huang LJ, Kuo SC, Wang JP (2005). Inhibition of lipopolysaccharide-induced expression of inducible nitric oxide synthase by phenolic (3E)-4-(2-hydroxyphenyl)but-3-en-2-one in RAW 264.7 macrophages. Biochem Pharmacol.

[B32] Zhao Q, Lee FS (1999). Mitogen-activated protein kinase/ERK kinase kinases 2 and 3 activate nuclear factor-κB through IκB kinase-α and IκB kinase-β. J Biol Chem.

[B33] Jeon YJ, Kim YK, Lee M, Park SMS, Han B, Kim HM (2000). Radicicol suppresses expression of inducible nitric-oxide synthase by blocking p38 kinase and nuclear factor-kappaB/Rel in lipopolysaccharide-stimulated macrophages. J Pharmacol Exp Ther.

[B34] Lee JS, Jung ES, Park JH, Jung KS, Lee SY, Hong ST, Park JN, Park EY, Kim JE, Park SH, Park DH (2005). Anti-inflammatory effects of Magnolol and Honokiol are mediated through inhibition of the downstream pathway of MEKK-1 in NF-B activation signaling. Planta Med.

[B35] Moon DO, Park SY, Heo MS, Kim KC, Park C, Ko WS, Choi YH, Kim GY (2006). Key regulators in bee venom-induced apoptosis are Bcl-2 and caspase-3 in human leukemic U937 cells through down regulation of ERK and Akt. Int Immunopharmacol.

[B36] Jang SI, Kim HJ, Kim YJ, Jeong SI, You YO (2006). Tanshinone IIA inhibits LPS-induced NF-kappaB activation in RAW 264.7 cells:possible involvement of the NIK-IKK, ERK1/2, p38 and JNK pathways. Eur J Pharmacol.

[B37] Son DJ, Ha SJ, Song HS, Lim Y, Yun YP, Lee JW, Moon DC, Park YH, Park BS, Song MJ, Hong JT (2006). Melittin inhibits vascular smooth muscle cell proliferation through induction of apoptosis via suppression of nuclear factor-kappaB and Akt activation and enhancement of apoptotic protein expression. J Pharmacol Exp Ther.

[B38] Minutoli L, Antonuccio P, Polito F, Bitto A, Fiumara T, Squadrito F, Nicotina PA, Arena S, Marini H, Romeo C, Altavilla D (2007). Involvement of mitogen-activated protein kinases (MAPKs) during testicular ischemia-reperfusion injury in nuclear factor-kappaB knock-out mice. Life Sci.

[B39] Sethi G, Ahn KS, Xia D, Kurie JM, Aggarwal BB (2007). Targeted deletion of MKK4 gene potentiates TNF-induced apoptosis through the down-regulation of NF-kappa B activation and NF-kappa B-regulated antiapoptotic gene products. J Immunol.

[B40] Jijon H, Allard B, Jobin C (2004). NF-kappaB inducing kinase activates NF-kappaB transcriptional activity independently of IkappaB kinase gamma through a p38 MAPK-dependent RelA phosphorylation pathway. Cell Signal.

[B41] de Haij S, Bakker AC, Geest RN van der, Haegeman G, Vanden Berghe W, Aarbiou J, Daha MR, van Kooten C (2005). NF-kappaB mediated IL-6 production by renal epithelial cells is regulated by c-jun NH2-terminal kinase. J Am Soc Nephrol.

[B42] Tuyt LM, Dokter WH, Birkenkamp K, Koopmans SB, Lummen C, Kruijer W, Vellenga E (1999). Extracellular-regulated kinase 1/2, Jun N-terminal kinase, and c-Jun are involved in NF-kappa B-dependent IL-6 expression in human monocytes. J Immunol.

[B43] Zhu J, Krishnegowda G, Gowda DC (2005). Induction of proinflammatory responses in macrophages by the glycosylphosphatidylinositols of Plasmodium falciparum: the requirement of extracellular signal-regulated kinase, p38, c-Jun N-terminal kinase and NF-kappaB pathways for the expression of proinflammatory cytokines and nitric oxide. J Biol Chem.

[B44] Warner TD, Mitchell JA (2004). Cyclooxygenases: new forms, new inhibitors, and lessons from the clinic. FASEB J.

[B45] Bertolini A, Ottani A, Sandrini M (2002). Selective COX-2 inhibitors and dual acting anti-inflammatory drugs: critical remarks. Curr Med Chem.

[B46] Nguyen HX, Tidball JG (2003). Expression of a muscle-specific, nitric oxide synthase transgene prevents muscle membrane injury and reduces muscle inflammation during modified muscle use in mice. J Physiol.

[B47] Abramson SB, Attur M, Amin AR, Clancy R (2001). Nitric oxide and inflammatory mediators in the perpetuation of osteoarthritis. Curr Rheumatol Rep.

[B48] Xie QW, Kashiwabara Y, Nathan C (1994). Role of transcription factor NF-kappa B/Rel in induction of nitric oxide synthase. J Biol Chem.

[B49] Baeuerle PA, Baltimore D (1996). NF-kappaB: ten years after. Cell.

